# Exploration of mRNA-modifying METTL3 oncogene as momentous prognostic biomarker responsible for colorectal cancer development

**DOI:** 10.1515/med-2025-1167

**Published:** 2025-03-26

**Authors:** Muhammad Naveed, Muhammad Saad Mughal, Tariq Aziz, Syeda Izma Makhdoom, Hamza Jamil, Ayaz Ali Khan, Nawal Al-Hoshani, Fakhria A. Al-Joufi, Roaa Mohammed Tahir Kassim, Maher S. Alwethaynani

**Affiliations:** Department of Biotechnology, Faculty of Science and Technology, University of Central Punjab, Lahore, 54590, Pakistan; Laboratory of Animal Health Food Hygiene and Quality, University of Ioannina, Arta, 47132, Greece; Department of Biotechnology, University of Malakand, Chakdara, 18800, Pakistan; Department of Biology, College of Science, Princess Nourah bint Abdulrahman University, Riyadh, 11671, Saudi Arabia; Department of Pharmacology, College of Pharmacy, Jouf University, 72341, Aljouf, Saudi Arabia; Department of Biology, College of Science, Umm Al-Qura University, Makkah, Saudi Arabia; Department of Clinical Laboratory Sciences, College of Applied Medical Sciences, Shaqra University, Alquwayiyah, Riyadh, Saudi Arabia

**Keywords:** colorectal, RMGs, biomarker, enrichment, immune cells, mutation, interaction

## Abstract

**Background:**

Colorectal cancer (CRC) is a leading cause of cancer-related mortality worldwide, emphasizing the need for improved prognostic biomarkers. Recent studies have identified the mRNA-modifying METTL3 oncogene as a potential biomarker in CRC progression.

**Objective:**

This study aimed to investigate the expression patterns of METTL3 in CRC, assess its association with clinical outcomes, identify interacting proteins and biological pathways, and explore its correlation with immune cell infiltration.

**Methods:**

Comprehensive analyses were conducted using public datasets, including transcriptome profiles from The Cancer Genome Atlas and the GSE103512 dataset. Protein–protein interaction (PPI) networks, pathway enrichment, and immune infiltration analyses were performed to elucidate METTL3’s role in CRC progression.

**Results:**

METTL3 expression was significantly higher in CRC tissues compared to normal tissues (*p* < 0.001). Mutations in METTL3 were detected in approximately 6% of CRC cases, with fusion events involving the SRPK2 gene. PPI analysis identified ten interacting proteins, including METTL4, EIF3H, RBM15B1, CBLL1, WTAP, NCBP1, RBM15, ZC3H13, METTL14, and KIAA1429. METTL3 expression showed a positive correlation with METTL4, METTL14, NCBP1, and WTAP expression (*R* > 0.5, *p* < 0.001). Higher METTL3 expression was associated with immunosuppressive phenotypes, such as increased infiltration of tumor-associated macrophages, regulatory T cells, and cancer-associated fibroblasts (*p* < 0.001). Pathway enrichment analysis revealed METTL3’s involvement in crucial pathways, including the cell cycle and renal cell carcinoma (*p* < 0.01). Gene ontology analysis highlighted its role in mRNA and RNA-related processes.

**Conclusion:**

The study supports the potential of METTL3 as a prognostic biomarker in CRC and highlights its involvement in immune modulation and cancer progression. These findings lay the groundwork for future studies aimed at developing targeted therapies and improving patient outcomes.

## Graphical abstract

**Figure:**
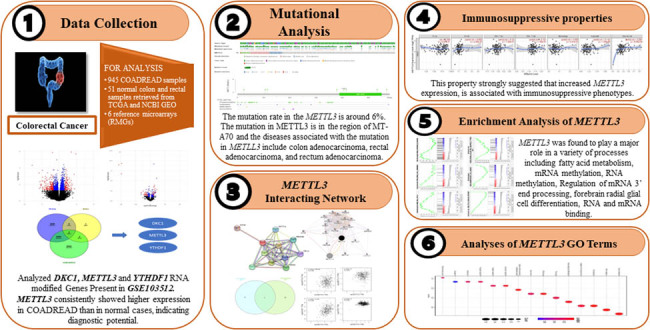

## 1 Introduction

Colorectal cancer (CRC) is a leading cause of cancer-related mortality worldwide, and its prognosis remains a major concern. Over the years, researchers have focused on identifying novel biomarkers that can improve early detection and prognostic prediction of CRC [1]. In recent studies, an emerging mRNA-modifying *METTL3* oncogene has garnered significant attention as a potential prognostic biomarker in the progression of CRC. This exciting development holds promise for revolutionizing the diagnosis, treatment, and management of this devastating disease [2,3]. According to the National Cancer Institute, the colon, which is the longest portion of the large intestine, and/or the rectum can both develop cancer (the last several inches of the large intestine before the anus). The frequency of colon and rectal cancer, which are prevalent malignant tumors of Aden epithelial origin, rises with age [4].

Between 2014 and 2018, the number of new cases of CRC in Americans over 50 years of age declined slightly yearly, but instances in Americans under 50 years of age significantly rose yearly [5]. Deaths from CRC decreased annually from 2015 to 2019 by a little amount. Men are diagnosed with CRC more frequently than women. The *METTL3* oncogene belongs to the METTL family of proteins and plays a crucial role in RNA methylation, a post-transcriptional modification process involved in gene expression regulation [6]. Recent investigations have revealed aberrant expression and dysregulation of *METTL3* in CRC, indicating its potential involvement in tumor progression. By catalyzing the addition of a methyl group to RNA molecules, *METTL3* influences mRNA stability, translation efficiency, and splicing patterns, ultimately impacting gene expression networks that drive cancer development [7].

Early studies have shown that aberrant *METTL3* expression levels correlate with clinical features such as tumor stage, metastasis, and overall survival rates in CRC patients [8]. These findings suggest that *METTL3* could serve as a reliable indicator of disease progression and patient outcomes. By identifying and monitoring *METTL3* expression patterns, clinicians may be able to make more informed decisions regarding treatment strategies, allowing for personalized therapies tailored to individual patients [9]. Transcriptomics has potential as a new method for accurately classifying cancer. In mammalian cells, gene expression silencing is a method of controlling transcription [10]. The process is carried out via RNA interference (RNAi), in which a short noncoding RNA joins an RNA-induced silencing complex and subsequently cleaves target mRNAs. Oncogenes and other driver genes important in cancer cell proliferation, the cell cycle, and tumor growth can be silenced by RNAi-mediated gene silencing, making it an interesting method for application in cancer treatment [11]. In this line, clinical trials are now assessing the therapeutic benefit from the use of short-interfering RNA-based therapies in cancer patients [12].

*DKC1* promotes the growth of CRC by stabilizing and binding to the mRNA of some ribosomal proteins in a manner that is reliant on its pseudo uridine synthase activity [13,14]. In this case, uridine is isomerized, resulting in the ribose being linked to C5 rather than the usual N1 in the process. Up to 100 pseudo uridine (“psi”) residues can be found in each rRNA; these residues may help to keep the structure of rRNAs stable, necessary for the preservation of telomeres and ribosome biosynthesis [15]. In addition, *YTHDF1* promotes both the ability for metastasis and proliferation of human colorectal cancer cells. It promotes the degradation of mRNAs that contain the amino acid m6A by interacting with the CCR4-NOT complex, acting as a regulator of mRNA stability. A number of genetic, epigenetic, and epitranscriptomic changes contribute to the development of colorectal tumors. The most prevalent messenger RNA (mRNA) modification in humans is RNA N6-methyladenosine (m6A), which plays a crucial role in posttranscriptional regulation of mRNA splicing, translation, and destruction [16].

The *METTL3*-*METTL14* heterodimer, an N6-methyltransferase complex, affects a number of functions, including the circadian clock, differentiation of embryonic and hematopoietic stem cells, cortical neurogenesis, response to DNA damage, T-cell differentiation, and initial miRNA processing [17]. The mechanistic role of *METTL3* in CRC progression could unveil new therapeutic targets and strategies. Researchers are actively investigating the downstream effects of *METTL3* dysregulation, exploring its impact on specific signaling pathways and biological processes that contribute to cancer growth and metastasis. By elucidating these mechanisms, scientists hope to develop targeted therapies that can modulate *METTL3* expression or its downstream effects, providing new avenues for treatment and potentially improving patient outcomes [18].

The aim of this study was to investigate the mRNA-modifying *METTL3* oncogene as a potential prognostic biomarker in CRC. The study employed a comprehensive approach that involved data collection from public repositories, differential expression analysis using transcriptome profiles from The Cancer Genome Atlas (TCGA) and GSE103512 datasets, gene expression analysis, protein–protein interaction (PPI) network analyses, mutation and correlation analyses, evaluation of *METTL3*’s role in immunosuppressive cells, and improvements in the quality of potential genes and pathways. By conducting these steps, the study aimed to determine the expression patterns of *METTL3* in CRC, assess its association with clinical outcomes, identify potential interacting proteins and biological pathways, and explore its correlation with immune cell infiltration. The findings from this study would contribute to understanding the significance of *METTL3* as a prognostic biomarker and its potential implications in CRC prognosis and treatment.

## 2 Materials and methods

### 2.1 Data collection

The keywords “RNA modification” were used to find the RMGs in PubMed. The datasets GSE103512 and TCGA, as well as the transcriptome profiles, were downloaded from Gene Expression Omnibus (GEO) (https://www.ncbi.nlm.nih.gCOADREAD/gds). The following analyses were conducted using 715 COADREAD and 15 normal cases in TCGA and a total of 64 COADREAD and 18 normal cases in GSE103512.

### 2.2 Differential expression analysis

The differentially expressed genes were found using the GEO2R, an interactive web tool that enables users to examine the gene expression between various groups of samples in a GEO dataset (DEGs). To examine TCGA expression profile of CRC, the R package “Deseq2” is used. The cut-off parameters for excluding DEGs were |log2FC| > 1 and a *p* value of 0.05. Table S1 contains a list of the DEGs from the two datasets. Then, using Venny 2.1, the RMGs and DEGs that overlapped between GSE103512 and TCGA were chosen and used for additional analysis.

### 2.3 Gene expression analysis using TCGA and GEO datasets

TCGA and GSE103512 expression profiling data were used to conduct the *METTL3* expression study. The efficacy of the expression level of any gene of interest in differentiating between COADREAD and healthy samples was then assessed using the webserver UALCAN (http://ualcan.path.uab.edu/index.html).

### 2.4 PPI network analyses

The functional PPIs of *METTL3* were found using the STRING database, which can predict and analyze functional interactions between proteins. Gene networks embracing *METTL3* were found using GeneMANIA (http://genemania.org/).

### 2.5 METTL3 mutation and correlation analyses

Using cBioPortal (https://www.cbioportal.org/), the *METTL3* mutation was carried out. Based on TCGA data, the GEPIA (Gene Expression Profiling Interactive Analysis) database (http://gepia.cancer-pku.cn/) was used to evaluate the *METTL3*-associated genes.

### 2.6 Evaluation of METTL3’s role in immunosuppressive cells

TIMER (https://cistrome.shinyapps.io/timer/) is a web-based program used in the study of the interplay between various cancers and the immune system. Using many gene markers for immune cell infiltration, we found that *METTL3* expression correlated with tumor infiltration by diverse immune cells. Multiple genetic markers were used to make this connection. The purity-adjusted partial Spearman’s rho value can be used to measure the strength of the association between immune cells and genes; a *p*-value of 0.05 indicates statistical significance.

### 2.7 Improvements in the quality of potential genes and pathways

We used gene set enrichment analysis (GSEA) to identify potential biological pathways involving *METTL3*. The study of the enriched gene set was judged significant when the nominal *p*-value was less than 0.01 and the false COADREAD rate (FDR) *q*-value was less than 0.05. cBioPortal study revealed that 16149 genes had a positive connection with *METTL3* (*R* > 0.3, *p* < 0.05), and these genes were used in the BP, CC, and MF studies. In light of these results, bubble diagrams were made using the “ggplot2” R Studio tool to display the findings of the BP, CC, and MF investigations.

## 3 Results

### 3.1 COADREAD DEGs-RMGs TCGA and GEO data

**Figure 1:**
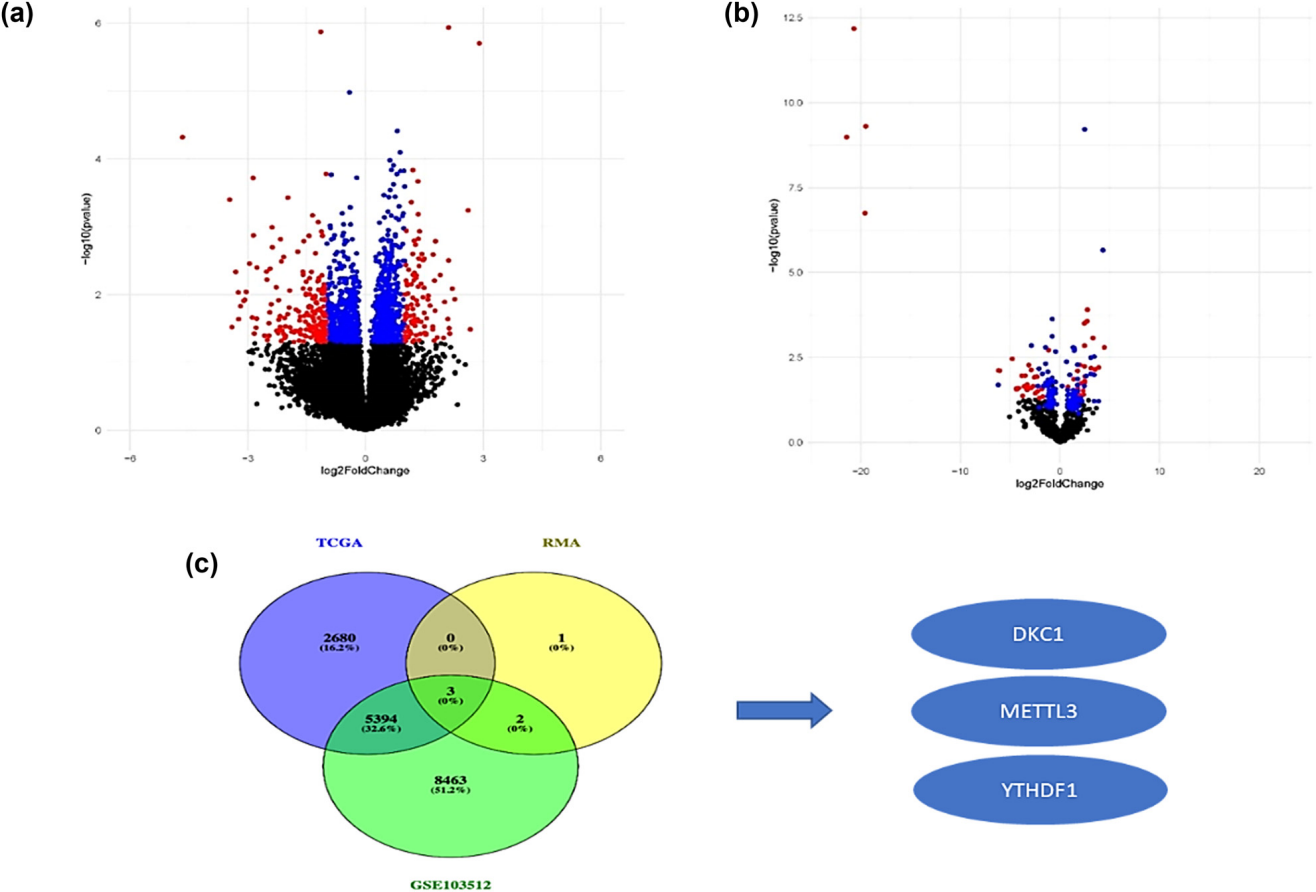
The identification of DEGs-RMGs using COADREAD data in TCGA and GEO. (a) and (b) The volcano plot of DEGs in GSE103512 and TCGA data. (c) DKC1, YTHDF1, and METTL3 were identified as the overlapping genes of DEGs in both datasets.

From PubMed, we selected a total of 6 RMGs. The GEO and UCSC Xena databases were mined for CRC in GSE103512 cohorts and TCGA AffyU133a expression profiles. Figure 1a displays DEGs obtained using GEO2R and R package “Deseq2” analyses between COADREAD and normal samples, and a total of 16,149 and 44,171 DEGs were obtained in TCGA dataset and the GSE103512 dataset, respectively (Table S1). By comparing the RMGs and DEGs from both groups, we were able to locate diagnostic biomarkers that could be employed across both groups. DKC1, YTHDF1, and *METTL3* were thus identified as RMGs according to their differential expression (Figure 1b). Due to the fact that DKC1 and YTHDF1 revealed contrasting patterns of expression in CRC (COADREAD) and normal tissues in TCGA and GSE103512 (Figure 1c). However, *METTL3* consistently showed higher expression in COADREAD than in normal cases, indicating diagnostic potential.

### 3.2 METTL3 in CRC: Mutation analysis

**Figure 2:**
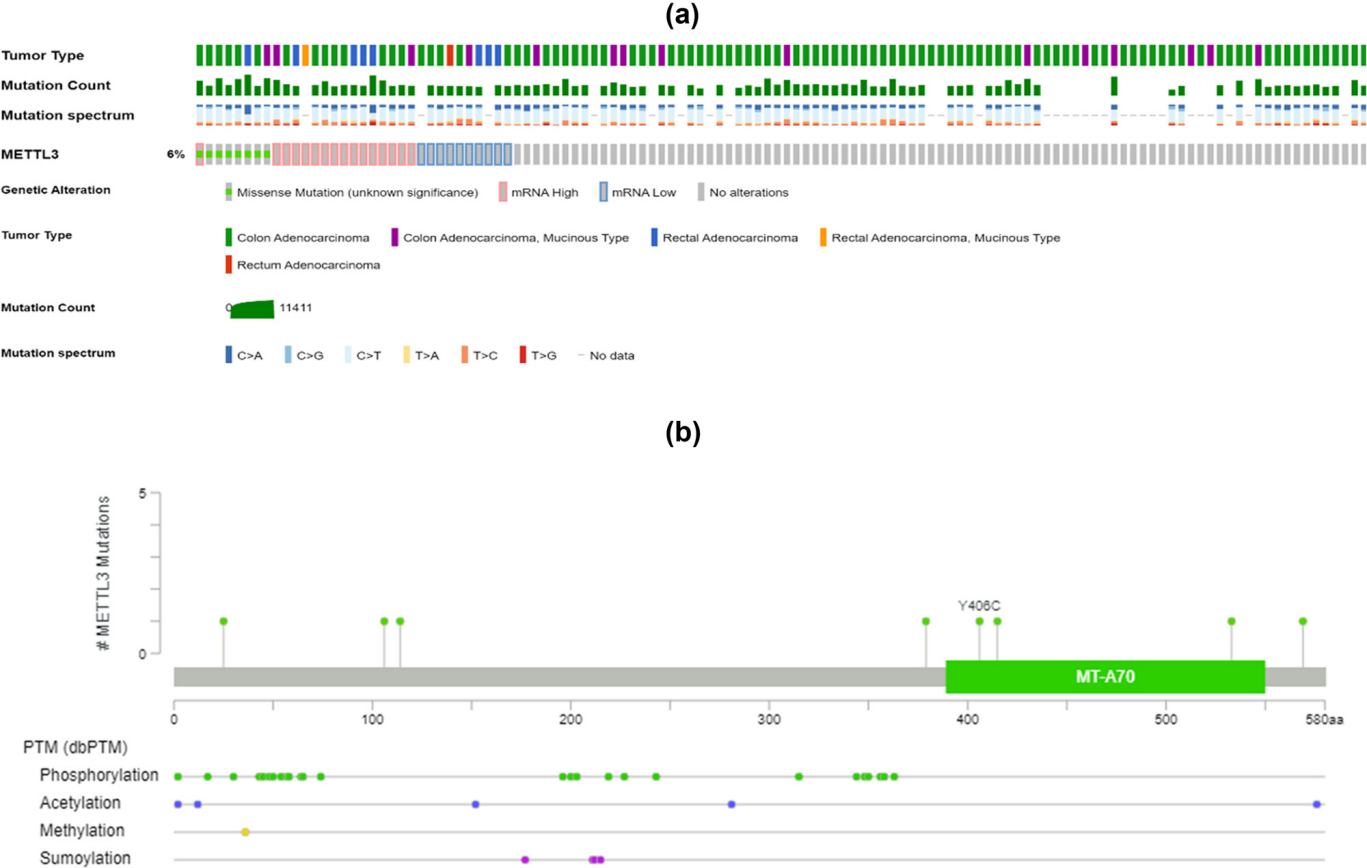
Mutation analysis in METTL3 gene. (a) Mutation type, spectrum, and genetic alteration in METTL3 gene. (b) DKC1, YTHDF1, and METTL3 were identified as the overlapping genes of DEGs in both datasets.

A mutation study of *METTL3* in cBioPortal revealed the fusion of *METTL3* with SRSF Protein Kinase 2 (SRPK2) in serous CRC, which was expected given the association between mutations in RNA modification genes and numerous forms of human malignancies (Table 1). The mutation rate in the *METTL3* is around 6% (Figure 2a). The mutation in *METTL3* is in the region of MT-A70 and the diseases associated with the mutation in METLL3 include colon adenocarcinoma, rectal adenocarcinoma, and rectum adenocarcinoma (Figure 2b).

**Table 1:** The mutation distribution of METTL3 in ovarian cancer according to cBioPortal

Study of origin	Sample ID	Cancer type detailed	Protein change	Mutation type	Variant type	Copy no.
CRC	TCGA-AD-6889-01	Colon adenocarcinoma	Y569C	Missense	SNP	Diploid
CRC	TCGA-G4-6309-01	Colon adenocarcinoma	T106M	Missense	SNP	Diploid
CRC	TCGA-A6-3807-01	Colon adenocarcinoma	D114G	Missense	SNP	ShallowDel
CRC	TCGA-AA-3989-01	Colon adenocarcinoma	Y406C	Missense	SNP	ShallowDel
CRC	TCGA-AA-3864-01	Colon adenocarcinoma	R415C	Missense	SNP	Diploid
CRC	TCGA-AG-A002-01	Rectal adenocarcinoma	L533S	Missense	SNP	Diploid
CRC	TCGA-CM-4747-01	Colon adenocarcinoma	R25S	Missense	SNP	ShallowDel
CRC	TCGA-WS-AB45-01	Mucinous adenocarcinoma of the colon and rectum	R379C	Missense	SNP	Diploid

### 3.3 METTL3-interacting network

**Figure 3:**
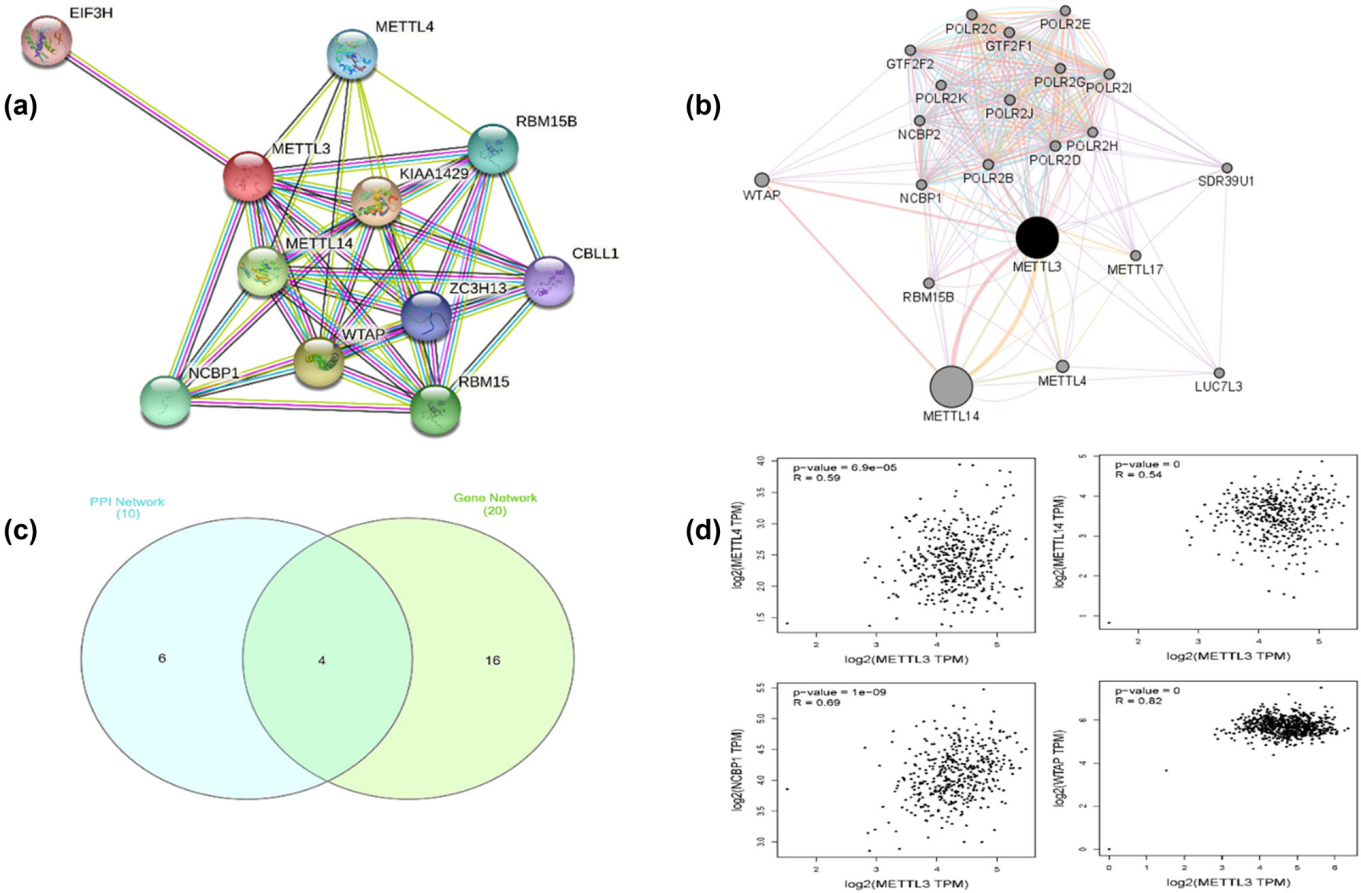
The interaction network and correlation of PUS7. (a) The protein interaction network of PUS7. Ten proteins *METTL4*, EIF3H, RBM15B1, CBLL1, *WTAP*, *NCBP1*, RBM15, ZC3H13, *METTL14*, and KIAA1429 physically/functionally interact with PUS7. (b) Twenty genes named *METTL14*, *WTAP*, *METTL4*, GTF2F2, POLR2D, RBM15B, GTF2F1, POLR2C, POLR2B, POLR2J, NCBP2, POLR2I, METTL17, *NCBP1*, LUC7L3, POLR2G, POLR2H, POLR2E, POLR2K, and SDR39U1 have physical interactions or genetic interactions, share protein domains with *METTL3*. (c) Four genes (*METTL14*, *METTL4*, *NCBP1*, and *WTAP*) were shared by the two networks. (d) The correlation analysis of *METTL3* with *METTL14*, *METTL4*, and *NCBP1.*

*METTL3* and its partner’s PPI and gene networks were analyzed using the String and GeneMANIA programs. According to the results of the PPI research, *METTL3* interacts with ten different proteins, including *METTL4*, EIF3H, RBM15B1, CBLL1, *WTAP*, *NCBP1*, RBM15, ZC3H13, *METTL14*, and KIAA1429 as shown in Figure 3a. Twenty genes (*METTL14*, *WTAP*, *METTL4*, GTF2F2, POLR2D, RBM15B, GTF2F1, POLR2C, POLR2B, POLR2J, NCBP2, POLR2I, METTL17, *NCBP1*, LUC7L3, POLR2G, POLR2H, POLR2E, POLR2K, and SDR39U1) were found in the GeneMANIA analysis to be physically or genetically associated with *METTL3* (Figure 3b). Four genes (*METTL14*, *METTL4*, *NCBP1*, and *WTAP*) were found to be common in both PPI and gene interaction (Figure 3c). Furthermore, GEPIA analysis demonstrated a strong correlation between *METTL3* expression and that of *METTL4* (*R* = 0.59, *p* = 6. 09 × 10^−5^), *METTL14* (*R* = 0.54, *p* = 0), *NCBP1* (*R* = 0.69, *p* = 1 × 10^−9^), and *WTAP* (*R* = 0.82, *p* = 0) as shown in Figure 3d.

### 3.4 Immunosuppressive properties in COADREAD tissues are associated with overexpression of METTL3

**Figure 4:**
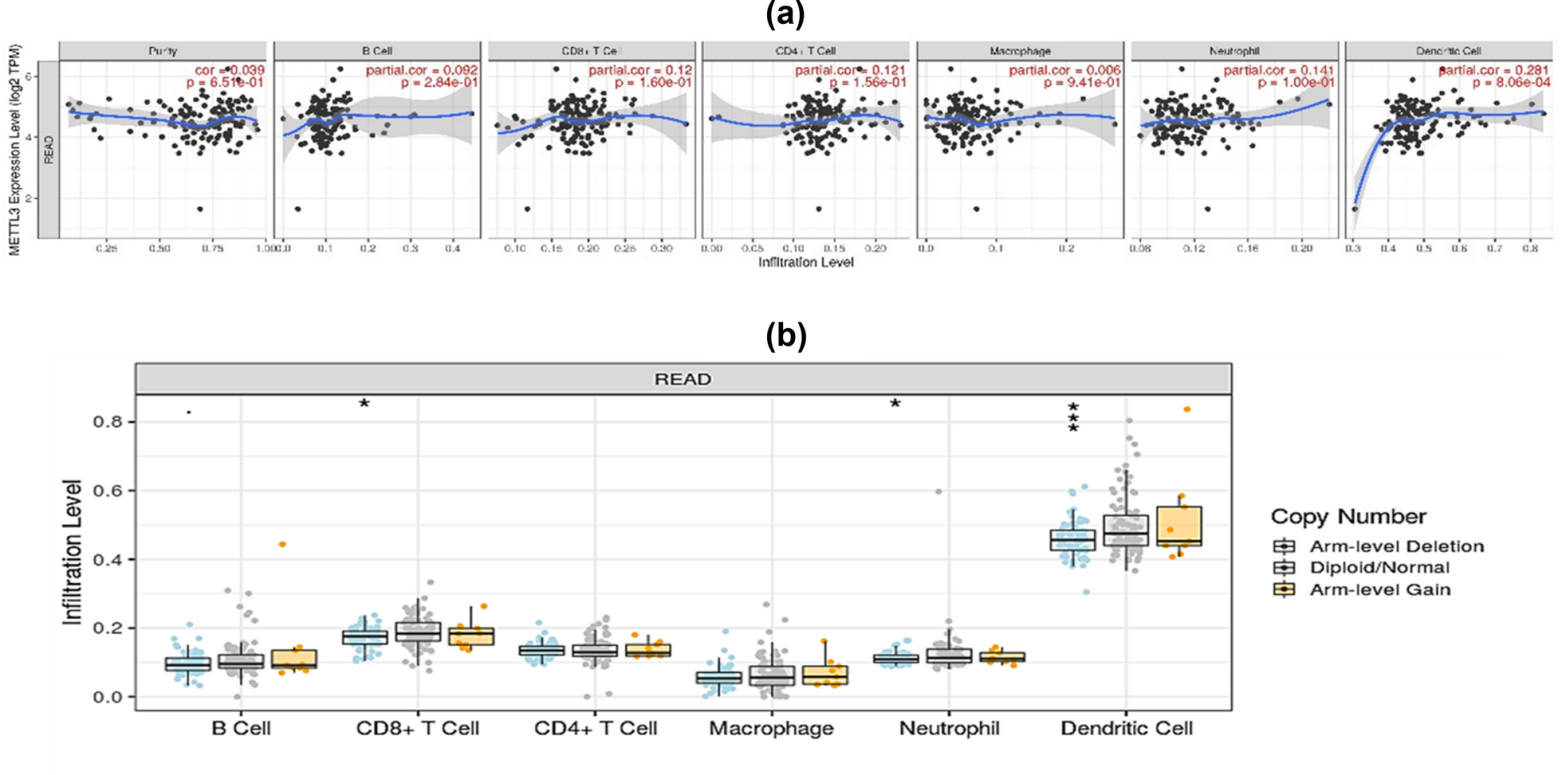
Immune infiltration analysis of *METTL3* gene within the tumor microenvironment (TME) of colon adenocarcinoma. (a) Expression level of different immune cells in COADREAD cancer. (b) Copy number of immune cells in READ only.

Here, we used TCGA cohorts to investigate whether high levels of COADREAD mRNA expression were associated with immunosuppressive cell infiltrations into tumors. We found an unexpected negative correlation between *METTL3* mRNA expression levels and tumor purity (Figure 4a). High levels of *METTL3* expression also correlate highly (all *p* < 0.001, cor > 0.3) with the infiltration levels of tumor-associated macrophages, regulatory T cells, and cancer-associated fibroblasts in bladder and prostate cancer cohorts. Conversely, it was discovered that the number of CD8+ T cells, a kind of antitumor T cell, infiltrated a tumor was inversely correlated with the number of *METTL3* mRNA copies (all *p* < 0.001, cor < 0) (Figure 4b). Combined, these data strongly suggested that increased *METTL3* expression, via a mechanism involving T-cell exclusion, is associated with immunosuppressive phenotypes in COADREAD tissues.

### 3.5 CRC: Pathway enrichment analysis of METTL3

**Figure 5:**
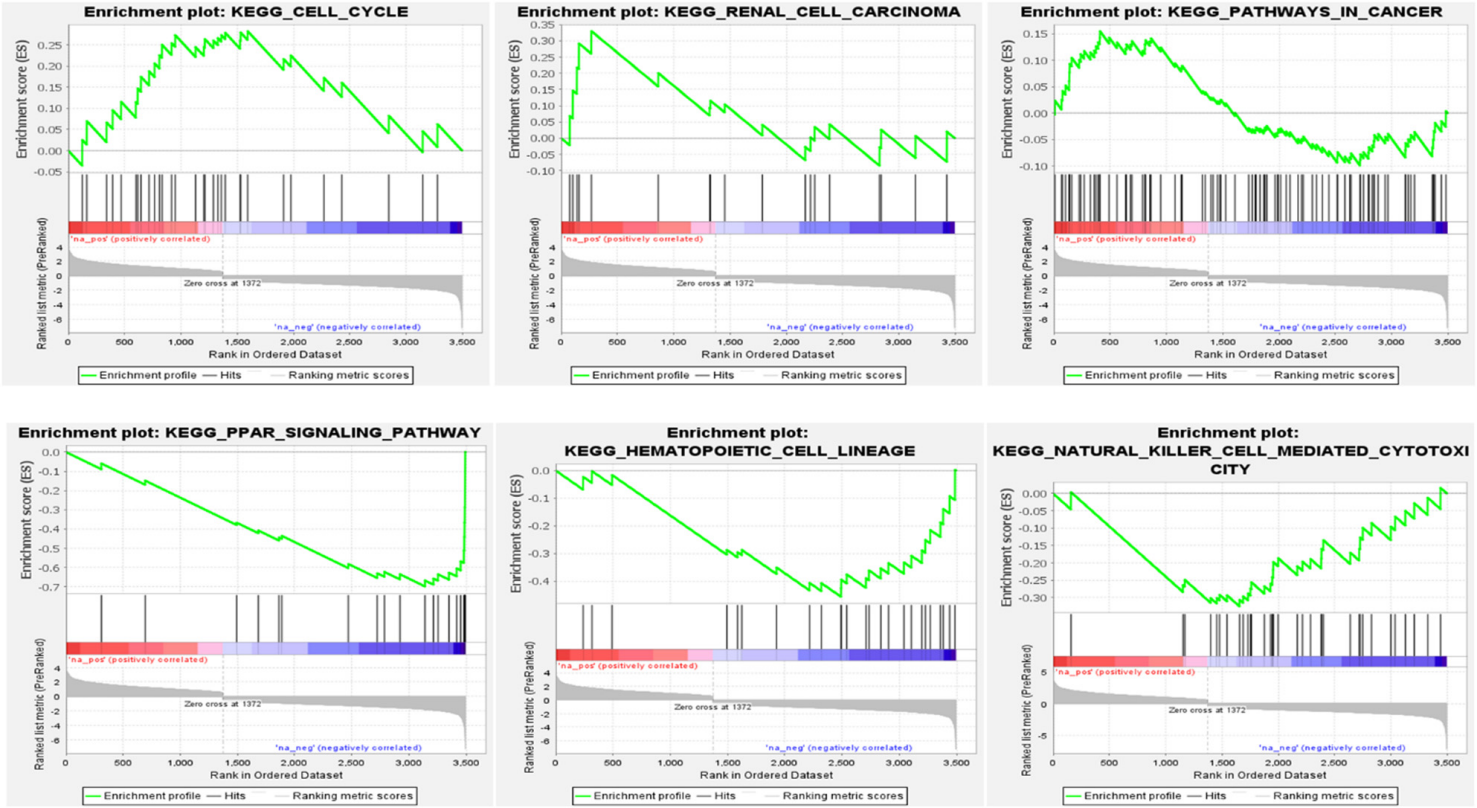
Top six pathway enrichment analyses of METTL3 in CRC.

A GSEA pathway analysis was carried out to investigate possible *METTL3*-related activities and regulatory processes in CRC. In CRC, *METTL3* is involved in six distinct pathways. Natural killer cell-mediated cytotoxicity, cell division, cancer, renal cell carcinoma, blood-forming stem cells, and PPAR signaling pathways are major pathways (Figure 5). Cell cycle, renal cell carcinoma, and pathways that are included in cancer stand out as two of the most crucial processes associated with the growth of CRC cells, among many others. These findings suggest that *METTL3* COADREAD expression in CRC may enhance tumor growth by modulating cell cycle progression and DNA replication.

### 3.6 Analyses of METTL3 GO terms in CRC

**Figure 6:**
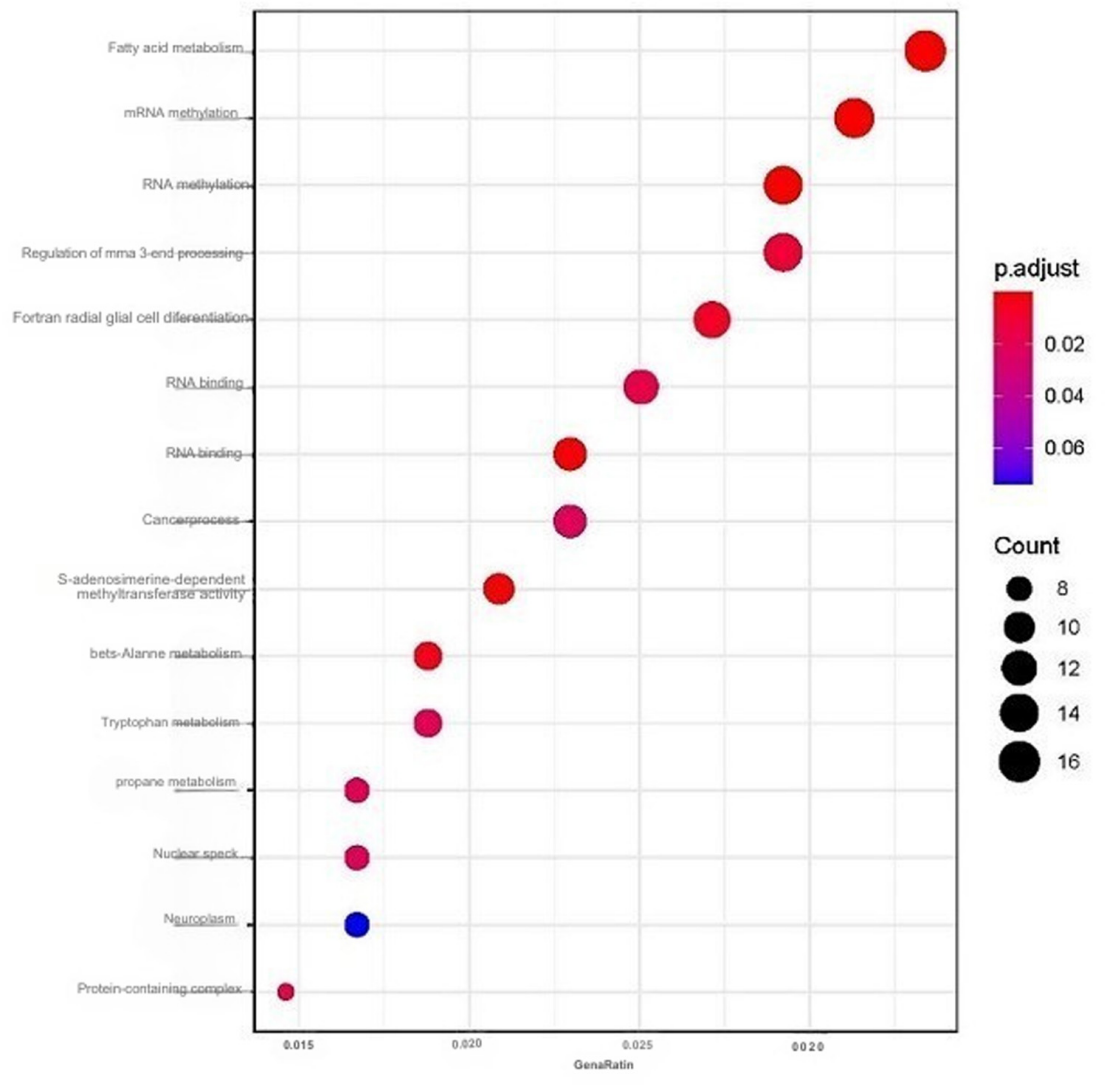
GO enrichment analysis of METTL3.

A total of 16,149 genes positively related to *METTL3* (*R* > 0.3, *p* < 0.0001) according to TCGA CRC data through the cBioPortal database were analyzed using R software, package “ggplot2” to further elucidate the GO enrichment analysis as shown in Figure 6. *METTL3* was found to play a major role in a variety of processes including fatty acid metabolism, mRNA methylation, RNA methylation, regulation of mRNA 3**′** end processing, forebrain radial glial cell differentiation, and RNA and mRNA binding.

## 4 Discussion

CRC is the third greatest cause of cancer-related death in the United States overall and the second leading cause among both men and women when data for both sexes are considered [18]. About 52,580 deaths are expected to occur in 2022 as a result. Too often, ovarian cancer is diagnosed after the disease has progressed significantly. The molecular pathways driving COADREAD metastases must be better understood before effective therapeutic options may be developed [19]. There are many obstacles in the way of effective treatment for this type of cancer, one of which is the search for diagnostic biomarkers. Researchers have found that most anti-cancer medications are cytotoxic, or destructive to all cells. Therefore, it is vital to develop a highly accurate diagnostic marker for COADREAD to enhance clinical diagnosis and raise the possibility of a longer life duration. Variations in RNA have been linked to a wide variety of human disorders, cancer included [20]. We used the RMG approach to identify the *METTL3* gene as a possible biomarker for CRC because there is currently no established biomarker for this disease [8]. In stem cells, *METTL3*-mediated pseudouridylation may “activate” some short RNAs produced from tRNAs to control protein synthesis and cell fate [21].

Our investigation into the deregulation of RMGs in CRC led us to *METTL3*, which is a one-of-a-kind potential biomarker for the detection of COADREAD [22]. We discovered the *METTL3* biomarker as a result of our research. This study has substantial clinical implications for the screening, prognosis, diagnosis, and staging of the disease in addition to treatment. The examination of gene expression reveals that patients with CRC (Sinicrope, 2022) had a higher level of METLL3 expression in comparison to healthy controls [23,24]. This validation adds credence to the concept of using METLL3 as a biomarker for the early diagnosis and prognostication of illness [25].

Tumor invasion, cancer growth, and the host immune response all rely on the intricate and ever-changing interactions between immune cells, the stoma, and cancer cells within the TME. Our study of immune infiltrating cells within the TME of the COADREAD tumor led us to the conclusion that high levels of METLL3 expression are correlated with high numbers of tumor-associated macrophages, regulatory T cells, and cancer-associated fibroblasts [6]. Tumors acquire an invasive character and T-cell exclusion as a result of immunosuppressive cells’ ability to dampen the activity of cytotoxic lymphocytes. Instead, we found a negative correlation between CD8+ T-cell immunological infiltration and *METTL3* mRNA expression, which suggests that elevated *METTL3* expression is associated with immunosuppressive features in COADREAD tissue via a mechanism involving T-cell exclusion [9].

Analysis of METTL3 as a prognostic biomarker in CRC has recently received extensive focus considering its various functions in tumorigenesis. Our study differs from previous works by using a multi-step approach which involves analyzing transcriptome data from TCGA and GSE103512 datasets, thereby leveraging on TIMER and GEPIA bioinformatics tools. Overall, this integration of multiple datasets and experimental methods can provide a better view of METTL3 in CRC development, immune infiltration, and the associated pathway enrichment, which are not well investigated in previous studies applying only individual dataset or single experimental approach [26].

Prior studies have only examined METTL3’s broad function in RNA methylation and its relevance in different cancers, such as lung and breast cancer. For example, Chen et al. showed that METTL3 contributed to CRC cell nutrition (GLUT1 Canal) and mTORC1 signal activation, making METTL3 a potential prognosis marker of CRC patients [27,28,29]. However, these studies rarely investigated the immune modulation functions of METTL3 in the TME, which is important for CRC progression. However, our results focus on the association between METTL3 and immunosuppressive subtypes, including TAM and Tregs, thus shedding new light on METTL3 as an immunosuppressive factor for CRC [30].

In the past, *METTL3* has never been linked to colon cancer. We investigated *METTL3*’s interaction partners to learn more about its function in COADREAD and how it promotes tumor detection, carcinogenesis, and growth [31,32]. *NCBP1* (Nuclear Cap Binding Protein Subunit 1) is a recently confirmed protein-coding gene that has been connected to a wide variety of diseases, including lung cancer. It is interconnected with the FGFR2 signaling in disease pathway and the mRNA splicing minor pathway. PPI and gene network analyses revealed novel interaction partners for *METTL3* in CRC, none of which had been described before. *WTAP* (WT1-associated protein) has been associated with Wilms tumors 1 and 5; however, no evidence has been found linking *WTAP* to cancer, indicating the unique nature of this protein interaction. The GSEA pathway study revealed that *METTL3* mainly regulates the cell cycle, renal cell carcinoma, and cancer pathways, which shed light on the *METTL3* signaling mechanism in CRC. These findings suggest that *METTL3* promotes the development of CRC by regulating pathways and the cell cycle. Still, further research is needed to verify this hypothesis.

The next steps in the study of METTL3 should lie in experimental and clinical applications of the bioinformatics data obtained. First, *in vitro* and *in vivo* experiments should confirm METTL3-regulated immune modulation and the identity of interacting TICs. Further mechanistic investigations are required to define the down-stream effects of METTL3 on immune cell recruitment and activity within the TME [33]. In addition, understanding the relationship between METTL3 and its reported protein targets in this list is relevant to CRC treatment (e.g., WTAP, METTL14) should be further investigated. Due to the increase in METTL3 research focusing on ovarian cancer, extending the study on METTL3 to other cancers that have similar immunosuppressive microenvironments would provide similar information for comparison [34,35]. Further, methods of functional genomics studying METTL3 may be enhanced by existing technologies like new-generation CRISPR-Cas9 gene editing and single-cell RNA sequencing. Preliminary identification of METTL3 small molecule inhibitors or RNA-based therapies may therefore open up new therapeutic avenues.

## 5 Conclusions

According to the findings of this study, *METTL3* is a novel RNA and protein biomarker for CRC that has the potential to be very beneficial. Further study has shown that *METTL3*, along with *METTL4*, *METTL14*, *NCBP1*, and *WTAP*, may interact to impact cell cycle, renal cell carcinoma, and pathways that are included in cancer and hence may influence CRC growth. The findings of the aforementioned investigations led to this discovery.

## Supplementary Material

Supplementary Table
